# Taxonomic annotation of public fungal ITS sequences from the built environment – a report from an April 10–11, 2017 workshop (Aberdeen, UK)

**DOI:** 10.3897/mycokeys.28.20887

**Published:** 2018-01-08

**Authors:** R. Henrik Nilsson, Andy F. S. Taylor, Rachel I. Adams, Christiane Baschien, Johan Bengtsson-Palme, Patrik Cangren, Claudia Coleine, Heide-Marie Daniel, Sydney I. Glassman, Yuuri Hirooka, Laszlo Irinyi, Reda Iršėnaitė, Pedro M. Martin-Sanchez, Wieland Meyer, Seung-Yoon Oh, Jose Paulo Sampaio, Keith A. Seifert, Frantisek Sklenář, Dirk Stubbe, Sung-Oui Suh, Richard Summerbell, Sten Svantesson, Martin Unterseher, Cobus M. Visagie, Michael Weiss, Joyce HC Woudenberg, Christian Wurzbacher, Silke Van den Wyngaert, Neriman Yilmaz, Andrey Yurkov, Urmas Kõljalg, Kessy Abarenkov

**Affiliations:** 1 Department of Biological and Environmental Sciences, University of Gothenburg, Box 463, 405 30 Göteborg, Sweden; 2 Gothenburg Global Biodiversity Centre, Box 461, SE-405 30 Göteborg, Sweden; 3 The James Hutton Institute and University of Aberdeen, Aberdeen, United Kingdom; 4 Plant and Microbial Biology, University of California, 94720 Berkeley, California, USA; 5 Leibniz Institute DSMZ-German Collection of Microorganisms and Cell Cultures, Inhoffenstrasse 7 B, 38124 Braunschweig, Germany; 6 Department of Infectious Diseases, Institute of Biomedicine, The Sahlgrenska Academy, University of Gothenburg, Guldhedsgatan 10, SE-413 46, Gothenburg, Sweden; 7 Department of Ecological and Biological Sciences, University of Tuscia, Viterbo 01100, Italy; 8 Department of Plant Pathology & Microbiology and Institute of Integrative Genome Biology, University of California, Riverside, Riverside 92501, CA, USA; 9 Université catholique de Louvain, Earth and Life Institute, Applied Microbiology, BCCM/MUCL, Louvain-la-Neuve, Belgium; 10 Department of Ecology and Evolutionary Biology, UC Irvine, Irvine, CA 92697, USA; 11 Department of Clinical Plant Science, Faculty of Bioscience, Hosei University, 3-7-2 Kajino-cho, Koganei, Tokyo Japan 184-8584; 12 Sydney Medical School-Westmead Hospital, Molecular Mycology Research Laboratory, Centre for Infectious Diseases and Microbiology, Sydney, Australia; 13 University of Sydney, Marie Bashir Institute for Infectious Diseases and Biosecurity, Sydney, Australia; 14 Westmead Institute for Medical Research, Westmead, Australia; 15 Institute of Botany, Nature Research Centre, Žaliųjų ežerų Str. 49, 08406 Vilnius, Lithuania; 16 Bundesanstalt für Materialforschung und -prüfung (BAM), Department 4. Materials & Environment, Unter den Eichen 87, 12205 Berlin, Germany; 17 School of Biological Sciences, Seoul National University, Seoul, Republic of Korea; 18 UCIBIO-REQUIMTE, DCV, Faculdade de Ciências e Tecnologia, Universidade Nova de Lisboa, 2829-516 Caparica, Portugal; 19 Biodiversity (Mycology), Ottawa Research and Development Centre, Agriculture & Agri-Food Canada, Ottawa, ON, Canada K1A 0C6; 20 Department of Biology, University of Ottawa, 30 Marie Curie Ottawa, ON, Canada, K1N 6N5; 21 Department of Botany, Faculty of Science, Charles University, Prague, Czech Republic; 22 Institute of Microbiology, Academy of Sciences of the Czech Republic, v.v.i, Prague, Czech Republic; 23 BCCM/IHEM, Scientific Institute of Public Health WIV-ISP, Juliette Wytsmanstraat 14, 1050 Brussels, Belgium; 24 ATCC, 10801 University Blvd., Manassas, Virginia 20110, USA; 25 Sporometrics, 219 Dufferin Street, Suite 20C, Toronto, Ontario Canada, M6K 1Y9; 26 Dalla Lana School of Public Health, University of Toronto, Health Sciences Building, 155 College Street, 6th floor, Toronto, Ontario Canada, M5T 3M7; 27 Evangelisches Schulzentrum Martinschule, Max-Planck-Str. 7, 17491 Greifswald, Germany; 28 Biosystematics Division, ARC-Plant Health and Protection, P/BagX134, Queenswood 0121, Pretoria, South Africa; 29 Steinbeis-Innovationszentrum, Organismische Mykologie und Mikrobiologie, Vor dem Kreuzberg 17, 72070 Tübingen, Germany; 30 Westerdijk Fungal Biodiversity Institute, Uppsalalaan 8, 3584 CT Utrecht, The Netherlands; 31 Department of Experimental Limnology, Leibniz-Institute of Freshwater Ecology and Inland Fisheries, Alte Fischerhuette 2, D-16775 Stechlin, Germany; 32 University of Tartu, Tartu, Estonia

**Keywords:** Indoor mycobiome, built environment, molecular identification, fungi, taxonomy, systematics, sequence annotation, metadata, open data

## Abstract

Recent DNA-based studies have shown that the built environment is surprisingly rich in fungi. These indoor fungi – whether transient visitors or more persistent residents – may hold clues to the rising levels of human allergies and other medical and building-related health problems observed globally. The taxonomic identity of these fungi is crucial in such pursuits. Molecular identification of the built mycobiome is no trivial undertaking, however, given the large number of unidentified, misidentified, and technically compromised fungal sequences in public sequence databases. In addition, the sequence metadata required to make informed taxonomic decisions – such as country and host/substrate of collection – are often lacking even from reference and ex-type sequences. Here we report on a taxonomic annotation workshop (April 10–11, 2017) organized at the James Hutton Institute/University of Aberdeen (UK) to facilitate reproducible studies of the built mycobiome. The 32 participants went through public fungal ITS barcode sequences related to the built mycobiome for taxonomic and nomenclatural correctness, technical quality, and metadata availability. A total of 19,508 changes – including 4,783 name changes, 14,121 metadata annotations, and the removal of 99 technically compromised sequences – were implemented in the UNITE database for molecular identification of fungi (https://unite.ut.ee/) and shared with a range of other databases and downstream resources. Among the genera that saw the largest number of changes were *Penicillium*, *Talaromyces*, *Cladosporium, Acremonium*, and *Alternaria*, all of them of significant importance in both culture-based and culture-independent surveys of the built environment.

## Introduction

The built environment presents dry, harsh conditions for fungal life, and traditional estimates of “indoor” fungi run in the low hundreds (Flannigan et al. 2011; Khan and Karuppayil 2012). General taxonomic progress and studies based on high-throughput sequencing (HTS) of amplicons are changing our view of the built environment as a biologically depauperate habitat. In a global study of indoor dust, Amend et al. (2010) found no less than 4,473 approximately species-level fungal operational taxonomic units (OTUs; Blaxter et al. 2005) distributed across more than 20 different fungal orders. Similarly, Adams et al. (2013) found 986 fungal OTUs from a homogeneous set of houses in a California family housing complex. The majority may represent outdoor fungi that drifted indoors as dead or dormant stages such as hyphal fragments, spores, or other propagules, but these stages must be considered as at least one aspect of the built mycobiome. Furthermore, niches not typically considered in indoor surveys, such as house plants (including Christmas trees), may harbour groups of fungi such as endophytes or mycorrhizae that are not typically considered part of the indoor mycobiome but would be detected by sensitive molecular techniques. Understanding the built mycobiome, therefore, becomes a matter of understanding a much larger number of species than those traditionally considered to form the core indoor fungi. Similarly, many of the common indoor species have been divided into numerous new species, thereby increasing the number of indoor species considerably (e.g., *Aspergillusversicolor* divided into ten new species (Jurjević et al. 2012), *Penicilliumchrysogenum* into five species (Houbraken et al. 2012), and *Wallemiasebi* into four species (Nguyen et al. 2015, Jančič et al. 2015)).

There is good reason to study the built mycobiome and the built microbiome at large (Nevalainen et al. 2015; Stamper et al. 2016; Adams et al. 2016). In damp dwellings an increased risk for health problems is apparent from epidemiological studies (WHO 2009). Indoor fungi and fungal particles are linked to a range of medical conditions, including asthma onset, allergies, and fatigue (Norbäck et al. 2016; Tischer et al. 2016). Fungi are a serious cause of decay of building materials, including recently introduced components such as composite wood products and various types of wall board, in the presence of sufficient moisture (Mensah-Attipoe et al. 2016; Meyer et al. 2016). Food spoilage or biodeterioration of textiles or other objects used for clothing, furniture, or carpeting in homes, hospitals, factories, and agricultural settings are also of concern (Benedict et al. 2016; Cardinale et al. 2017; Garnier et al. 2017). This puts fungi on the research agenda for a range of scientific disciplines in addition to traditional mycology, which increases the pressure on mycology to produce data, results, and resources that are straightforward to apply for mycologists and non-mycologists alike. Most mycologists would presumably agree that you should not have to be a taxonomist or even a mycologist to be able to identity fungal DNA sequences from the built environment to a meaningful taxonomic level, such as genus or preferably species. Similarly, it should be straightforward also for non-mycologists to retrieve all public fungal DNA sequences collected on, say, interior walls or floors for further study. In reality, neither of these possibilities is feasible (cf. Abarenkov et al. 2016).

Molecular identification of fungi is largely centred on the nuclear ribosomal internal transcribed spacer (ITS) region, which is the formal fungal DNA barcoding marker (Schoch et al. 2012). However, a number of problems beset ITS-based molecular identification of fungi. To begin with, reference ITS sequences are available for less than 1% of the estimated 6 million extant species of fungi (Blackwell 2011; Taylor et al. 2014). This is coupled with the fact that some 50% of the ~750,000 fungal ITS sequences in the International Nucleotide Sequence Database Collaboration (INSDC: GenBank, ENA, and DDBJ; Cochrane et al. 2016) are not identified to species level, and of the sequences that do have a full Latin binomial, more than 10% may have names that are incorrect (Nilsson et al. 2012). On top of that, technical artefacts such as chimeric unions, poor sequence trimming, and low read quality are common (Kang et al. 2010; Hyde et al. 2013; Nilsson et al. 2017). Finally, many researchers are in the habit of submitting their sequences with very little associated metadata (such as country and substrate or host of collection), leaving more than 50% of the fungal ITS entries in the INSDC more or less non-attributable (Tedersoo et al. 2011). Taken together, these issues often make informed molecular identification of fungi difficult even for well-trained mycologists. It is therefore no surprise that non-mycologists struggle even more.

The UNITE database for molecular identification of fungi (https://unite.ut.ee/; Kõljalg et al. 2013) was designed to overcome these complications, with the ultimate purpose to offer robust and reproducible identification and reference to all species of fungi, whether or not formally described. UNITE draws from the public fungal ITS sequences in the INSDC and is centred on the concept of species hypotheses (SHs), which are approximately species-level OTUs derived from sequence clustering (Kõljalg et al. 2013). All SHs have unique, individual digital object identifiers (DOIs; https://www.doi.org/) for unambiguous reference across time and scientific studies (e.g., 10.15156/BIO/SH216455.07FU which resolves to http://dx.doi.org/10.15156/BIO/SH216455.07FU). UNITE recently received an Alfred P. Sloan Foundation grant to improve the support within the database for handling, processing, and characterising the mycobiome from the built environment (the built mycobiome). Several actions have been taken towards that goal, including a workshop to annotate all extant built-environment fungal ITS sequences according to the MIxS-BE annotation standard (Glass et al. 2014; Abarenkov et al. 2016). The present paper reports on the outcome of a taxonomic annotation workshop that specifically addressed fungal taxa and sequences from the built environment from taxonomic and nomenclatural points of view.

The workshop was held at the James Hutton Institute/University of Aberdeen on April 10–11 2017, and comprised 19 *in situ* and 12 remote participants. Fifteen of the participants had a taxonomic background and were tasked with assessing the public fungal ITS sequences and SHs within their respective expertise area in relation to assigned names, nomenclature, and recent taxonomic progress. Four of the participants had a general background in built-environment mycology and were asked to annotate recent sequences from the built environment according to the MIxS-BE standard. Nine participants had a background in other fields of mycology and were asked to harvest missing sequence metadata from the literature for fungal groups relevant to the built environment. Finally, four participants had a background in bioinformatics and were asked to process the corpus of public fungal ITS sequences from a technical point of view. All participants operated under the expectation that their contribution should meet the highest of quality standards, and that their work would be incorporated in UNITE, adopted by the downstream resources that make use of UNITE data (see, e.g., https://unite.ut.ee/repository.php), and shared with the INSDC and the recently established ISHAM database, which is a comprehensive, expertly curated ITS database of clinically important fungal pathogens (Irinyi et al. 2015).

## Materials and methods

### Taxonomic annotation of fungi related to the built environment

The participants examined the public sequences from their respective fungal groups of expertise from nomenclatural and taxonomic points of view through the PlutoF workbench of UNITE (Abarenkov et al. 2010). Sequences were given (or re-annotated with) names that reflected the level at which the taxonomist felt comfortable providing a name. Thus, some sequences were demoted from species level to genus level, some sequences were promoted from kingdom-level (“Uncultured fungus”) to order level (“Dothideales”), and so on. Other sequences were re-named to account for, e.g., recent synonymies and merger of anamorphic and teleomorphic names. From a taxonomic standpoint, reference sequences for individual SHs were designated at the similarity level (97–100% similarity) at which the application made the most taxonomic sense. Inter-specific divergence is known to be very low or even non-existent for the ITS region of certain species complexes or genera, for example parts of *Penicillium*, *Fusarium*, *Aspergillus*, and *Talaromyces* (Visagie et al. 2014; O’Donnell et al. 2015; Visagie et al. 2017; Yilmaz et al. 2014), and the participants sought to represent the species level as closely as possible for each SH, at times drawing from information from other genetic markers. The participants used recent publications, Index Fungorum (http://www.indexfungorum.org/), MycoBank (http://www.mycobank.org/), and other resources in this pursuit. In addition, all type-derived sequences from the NCBI RefSeq Targeted Loci Project (https://www.ncbi.nlm.nih.gov/refseq/targetedloci/; O’Leary et al. 2015) were considered during the workshop, and were designated as reference sequences for the corresponding SHs whenever possible.

### Annotation of built-environment sequences according to the MIxS-BE standard

Abarenkov et al. (2016) annotated all published, public fungal ITS sequences from the built environment – identifiable as such – according to one or more aspect of the MIxS-BE annotation standard. During the ~12 months that had elapsed after the Abarenkov et al. (2016) workshop, 924 new Sanger-derived fungal ITS sequences from the built environment had become available in the INSDC. Those 924 sequences, in so far as they corresponded to a formally published (or otherwise available) study, were examined and annotated according to the MIxS-BE standard following Abarenkov et al. (2016).

### Metadata assembly for sequences from species with a relation to the built environment

In UNITE, all sequences that are at least 80% similar are grouped into compound clusters, which are further clustered into SHs (Kõljalg et al. 2013). SHs that contain at least one sequence recovered from the built environment, regardless of whether or not the underlying species is known as a “traditional” indoor fungus, were considered for this task. Those SHs were examined for sequences that lacked explicit specification of both country of collection and host of collection. UNITE was found to contain more than 5,000 such sequences, and the workshop participants sought to reduce this number by applying country and/or host of collection to these entries through scrutiny of the underlying scientific publications (as available) or other online resources. These basic metadata were restored with the hope that the amalgamated information would assist present and future researchers in the interpretation of the biology of fungi with a relation to the built environment. Country names were specified according to the ISO 3166 standard. Hosts were specified by Latin names following the PlutoF consensus classification (Abarenkov et al. 2010).

### Analysis of sequences from a technical, quality-related point of view

Several of the workshop participants had a background in bioinformatics and focused on quality-related aspects of public fungal ITS sequences with and without a direct relation to the built environment. Chimera control was done following Nilsson et al. (2015, 2016), and sequence trimming/read quality was examined following Hyde et al. (2013) and Nilsson et al. (2017).

## Results

### Taxonomic annotation of fungi related to the built environment

The names of 4,783 sequences from a total of 387 distinct SHs were updated during the workshop (Supplementary material 1). A total of 505 reference sequences were established, nearly all of which were from type-derived material (and 21 of which stemmed from the built environment): 36 at the 97% similarity level, 39 at 97.5%, 46 at 98%, 62 at 98.5%, 83 at 99%, 103 at 99.5%, and 136 at 100%. The 10 genera that saw the greatest number of changes (name changes + reference sequence designations) are listed in Table 1. The results of the taxonomic annotation part of the workshop are summarized in Table 2.

**Table 1.** Overview of genera. The 10 genera that saw the largest number of taxonomic changes during the workshop, plus the number of such changes.

Genus	Number of changes
*Penicillium*	714
*Talaromyces*	601
*Cladosporium*	533
*Mortierella*	372
*Phialocephala*	327
*Funneliformis*	196
*Cyphellophora*	167
*Acremonium*	136
*Alternaria*	132
*Leohumicola*	106
Total	3284

**Table 2.** Results of the taxonomic annotation part of the workshop. Name updates = number of sequences whose names were updated. RefS designations = number of reference sequences designated for individual SHs. Chimeras = number of chimeric sequences identified. Low read quality = number of sequences marked as being of substandard technical quality. The chimeras and the low read quality sequences were excluded from further use in UNITE (although kept in the system for future reference). Studies = number of distinct studies that saw at least one change to at least one sequence.

	Name updates	RefS designations	Chimeras	Low read quality	Sum of changes	Studies
Sequences	4783	505	5	94	5387	250

### Annotation of built-environment sequences according to the MIxS-BE standard

**Figure 1.**
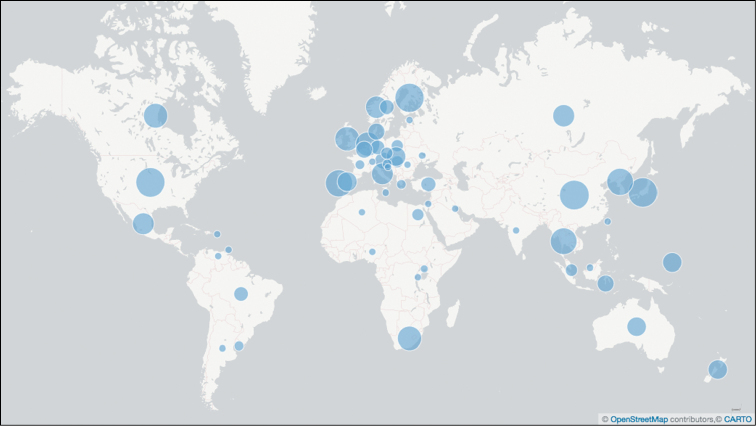
Analysis of the built environment sequences for country of collection. Country centroids based on the geographical centres of contiguous country land masses are marked with bubbles of different size on the global map to indicate the number of built environment sequences originating from these countries as stated explicitly in the underlying INSDC records or as restored during the present effort and in Abarenkov et al. (2016) (57 distinct countries, sequence count ranging from 1 to 3,091). The figure is based on Abarenkov et al. (2016) plus the data added during the workshop, such that it indicates the scientific state of ITS-based Sanger-derived sequencing of the built mycobiome as of spring 2017.

**Figure 2.**
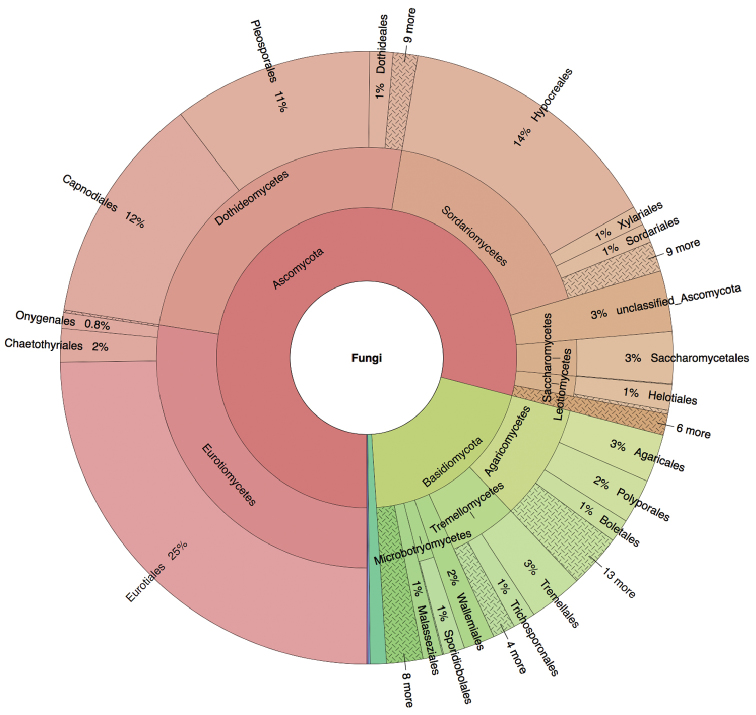
Krona chart of the taxonomic affiliation of the built environment sequences down to order level. The Krona chart lists all annotated built environment sequences except those classified as Fungi sp. (32%) and those of non-fungal origin (1%). An interactive version of the Krona chart is provided as Supplementary material 4. The figure is based on Abarenkov et al. (2016) plus the data added during the workshop, such that it indicates the scientific state of ITS-based Sanger-derived sequencing of the built mycobiome as of spring 2017.

**Figure 3.**
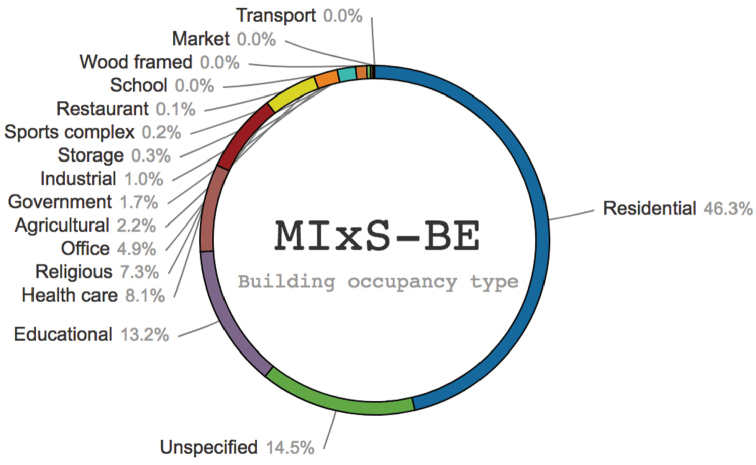
Analysis of the MIxS-BE “building occupancy type” (type of building where the underlying sample was taken). The figure is based on Abarenkov et al. (2016) plus the data added during the workshop, such that it indicates the scientific state of ITS-based Sanger-derived sequencing of the built mycobiome as of spring 2017.

A total of 922 of the 924 sequences from the built environment – corresponding to 33 different studies deposited since Abarenkov et al. (2016) – were annotated with at least one MIxS-BE metadata item during the workshop (Table 3). A total of 1,848 MIxS-BE annotations were made during the workshop. For example, “building occupancy type” was established for 597 sequences, and “indoor surface” was established for 76 sequences. Analyses of the geographical, taxonomic, and “building occupancy type” origin of all fungal ITS sequences from the built environment are provided in Figures 1–3. These figures are based on Abarenkov et al. (2016), to which the results of the present workshop (Supplementary material 2) were added.

**Table 3.** Results of the metadata annotation part of the workshop, specified for the built mycobiome sequence set (BMS) and the outdoor mycobiome sequence set (OMS). Country and host of collection plus host association were assembled for both of these. The number of sequences processed, plus the number of underlying published and unpublished scientific studies, are also provided. For the BMS, the nine MIxS-BE annotation standard items targeted at the workshop are specified in separate columns. The sequence numbers shown in the table refer to the number of sequences annotated for each data item.

	Number of sequences (annotated)	Number of different studies	Country of collection	Different countries	Host of collection	Different hosts	Host association	Comment	
BMS	924 (922)	33	543	10	218	2	218	865	
OMS	7657 (5264)	218	4452	84	1524	275	1272	3181	
Both jointly	8581 (6186)	250	4995	84	1742	276	1490	4046	
	build_occup_type	space_typ_state	substructure_type	ventilation_type	indoor_space	indoor_surf	surf_material	surface-air contaminant	filter_type
BMS	597	732	19	95	4	76	130	195	0

### Metadata assembly for sequences from species with a relation to the built environment

A total of 5,264 sequences from a total of 218 distinct studies were annotated with at least one metadata item. A total of 10,429 metadata annotations were made during the workshop, including 4,452 country of collection (84 distinct countries) and 1,524 host of collection (275 distinct hosts; Table 3; Supplementary material 3).

### Analysis of sequences from a technical, quality-related point of view

Five sequences were marked for removal from the SH system because they were chimeric. Another 94 sequences were marked for removal because of low read quality.

## Discussion

Jointly the workshop participants implemented a total of 19,508 changes in UNITE (Tables 2–3). Some 27% (5,288) were taxonomically-related in the sense of giving sequences correct names or designating reference sequences (and their similarity threshold of application) for SHs. Although these numbers may sound impressive, several participants reported that they were unable to finish the annotation of their genera of expertise. In many cases, these genera contained dozens to hundreds of species, highlighting the very substantial amount of time required to process them. Indeed, for the larger genera, no single taxonomic expert can be expected to know all species equally well, and no single researcher can be expected to oversee the annotation of the entire genus. Input from different researchers is clearly needed to process large genera such as *Penicillium*, *Aspergillus*, and *Fusarium*. The workshop participants were asked to focus on the species they knew well, and this report makes no claim of exhaustiveness regarding the genera covered during the workshop or fungi in the built environment in general.

Several participants expressed frustration over the fact that numerous scientific studies were found to have released hundreds of sequences identified only as “Uncultured fungus” (or similar) even when a more informative name would be only seconds away through, e.g., a BLAST search (Altschul et al. 1997). The two presumed main reasons why a researcher would deposit a sequence under the name “Uncultured fungus” would be lack of time to investigate the taxonomic affiliation of the sequence prior to deposition, and concerns about providing a Latin name that would later turn out to be incorrect. As far as fungal sequences go, “Uncultured fungus” will always be a correct – albeit very uninformative – name, and while we agree that it is an error-proof way of giving names to sequences, it also introduces uncertainty, especially for least recent ancestor analyses, and serves to mask fully identified reference sequences, much to the damage of molecular identification of fungi. Consider the case of a cloning-based study that gives rise to, say, 25 near-identical public sequences called “Uncultured fungus”. Another researcher happens to have the same species in a sample, generates an ITS sequence from that species, and uses that sequence for a BLAST search. Even if, say, two highly similar, non-cloning-based sequences with a full species name were available, the resulting BLAST output would be confusing (perhaps starting with 10–25 “Uncultured fungus” sequences as top matches). No wonder, then, that such a user might adopt the name “Uncultured fungus” for the new sequence, with the effect that uncertainty and mistakes will persist and may even be amplified over time (Gilks et al. 2002). The subsequent scientific study of that researcher would be plagued by yet another OTU not assigned beyond the kingdom level, needlessly depriving the study of fungi and fungal communities of much-needed resolution. We advocate that sequence depositors in the INSDC try to go beyond the kingdom level when assigning names to their newly generated sequences, at least for straightforward cases. This task should be undertaken by somebody with significant understanding of fungal systematics and sequence analysis. Instead of arguing that the taxonomic expertise to make such taxonomic calls were not available to the project team, researchers should plan their projects to include sufficient taxonomic expertise that the process of making such calls is feasible. We furthermore ask journal editors and reviewers to set high standards regarding the taxonomic annotations in any manuscript they handle.

Another issue that surfaced repeatedly during the workshop was the occurrence of legacy names, some of them downright outrageously outdated, and other obsolete data. In one case, a name that was synonymized more than 20 years ago was found. We take this to indicate that many researchers do not feel a personal responsibility for their INSDC submissions once those have become a part of the public corpus. However, this view goes against the INSDC policies (https://www.ncbi.nlm.nih.gov/genbank/submit/), which make it clear that sequence authors should approach the INSDC whenever additional explanatory information pertinent to their entries becomes available. Major changes to INSDC entries, such as changes in species names or the very sequence data, will also reach UNITE automatically. We hope that this workshop will serve as a general call to taxonomists and other researchers to revisit their previous INSDC submissions to see if they can be updated or if additional data can be provided. At an altruistic level, any such additional data are likely to move the study of fungi forward – in whatever context they are found – which should be at the heart of every mycologist. At a more personal level, researchers who ensure that “their” group of fungi are properly annotated in the public sequence databases, will soon start to see additional sequences for “their” fungi being identified and deposited by other researchers. This should translate into new opportunities for knowledge expansion and scientific collaboration, to the benefit of the initial researcher and, ultimately, everyone else.

The workshop also identified several shortcomings and avenues for improvement of the UNITE database. For example, recent taxonomic progress in fungi traditionally classified in the polyphyletic genera *Candida, Cryptococcus*, and *Rhodotorula* resulted in the recognition of a number of new genera and species names (e.g., Daniel et al. 2014; Kurtzman 2014; Liu et al. 2015; Wang et al. 2015). These changes necessitate the renaming of hundreds to thousands of sequences at, typically, the genus level. At present, UNITE has no software support for batch renaming of sequences at the SH or genus levels, suggesting an urgent need for improvement of UNITE. Similarly, several workshop participants expressed the need to name sequences according to publications that were “in press” or that were published just weeks or a month ago. UNITE, and to some extent Index Fungorum/MycoBank, operate at longer time scales than weeks and will not always have the very latest information at hand. In a digital age where information is created and disseminated more or less simultaneously, updating information on a bimonthly basis is no longer sufficient. This observation puts pressure on UNITE to improve the frequency at which information is exchanged with Index Fungorum and other databases. A significant amount of work remains in terms of information exchange policies across databases such as the INDSC, UNITE, Index Fungorum, MycoBank, and ISHAM-ITS. A change implemented in one of these databases does not necessarily reach the others. As one participant pointed out, it is frustrating enough to provide updates and corrections once for other researchers’ data. To have to do it twice, to two different repositories at that, is disheartening – and ultimately unlikely to occur. A solution to this problem would be the establishment of an integrated cloud-based dynamic database network that would allow an instantaneous update in all relevant databases. Towards that end, all changes implemented during this workshop were shared with the INSDC and ISHAM-ITS, and several updates were sent to Index Fungorum.

In conclusion, the present workshop implemented a total of 19,508 changes in UNITE relating to fungi in the built environment. This will undoubtedly improve the taxonomic resolution in studies of the built, as well as many other, mycobiomes. Although truly uncharacterized lineages of fungi are repeatedly found in the built environment (e.g., Nilsson et al. 2016), in many cases it is more likely lack of input from the mycological community that is responsible for the low taxonomic resolution that haunts many molecular ecology studies of fungal communities. If all taxonomic experts were to look through and annotate fungi in their areas of expertise in the international nucleotide sequence databases, the problem would be greatly diminished. However, it is not just expert taxonomists who can make a difference – sequence authors, article co-authors, reviewers, and editors should make it a habit to insist that sequence data are annotated beyond the barest minimum and in compliance with recent taxonomic progress and relevant metadata standards. An increasing number of non-mycologists now sequence fungi and fungal communities as a part of their professional pursuits, and it would greatly benefit mycology if these non-mycologists could obtain unambiguous, correct, and reproducible results.

## Supplementary materials

Supplementary material 1 The sequences renamed during the workshop. The INSDC accession number, the original INSDC name, and the new UNITE name are shown Data type: molecular data This is an open access article distributed under the terms of the Creative Commons Attribution License (CC BY 4.0), which permits unrestricted use, distribution, and reproduction in any medium, provided the original author and source are credited.R. Henrik Nilsson, Andy F. S. Taylor, Rachel I. Adams, Christiane Baschien, Johan Bengtsson-Palme, Patrik Cangren, Claudia Coleine, Heide-Marie Daniel, Sydney I. Glassman, Yuuri Hirooka, Laszlo Irinyi, Reda Iršėnaitė, Pedro M. Martin-Sanchez, Wieland Meyer, Seung-Yoon Oh, Jose Paulo Sampaio, Keith A. Seifert, Frantisek Sklenář, Dirk Stubbe, Sung-Oui Suh, Richard Summerbell, Sten Svantesson, Martin Unterseher, Cobus M. Visagie, Michael Weiss, Joyce Woudenberg, Christian Wurzbacher, Silke Van den Wyngaert, Neriman Yilmaz, Andrey Yurkov, Urmas Kõljalg, Kessy Abarenkov

Supplementary material 2 The MIxS-BE annotations implemented for the built environment sequences during the workshop Data type: molecular data This is an open access article distributed under the terms of the Creative Commons Attribution License (CC BY 4.0), which permits unrestricted use, distribution, and reproduction in any medium, provided the original author and source are credited.R. Henrik Nilsson, Andy F. S. Taylor, Rachel I. Adams, Christiane Baschien, Johan Bengtsson-Palme, Patrik Cangren, Claudia Coleine, Heide-Marie Daniel, Sydney I. Glassman, Yuuri Hirooka, Laszlo Irinyi, Reda Iršėnaitė, Pedro M. Martin-Sanchez, Wieland Meyer, Seung-Yoon Oh, Jose Paulo Sampaio, Keith A. Seifert, Frantisek Sklenář, Dirk Stubbe, Sung-Oui Suh, Richard Summerbell, Sten Svantesson, Martin Unterseher, Cobus M. Visagie, Michael Weiss, Joyce Woudenberg, Christian Wurzbacher, Silke Van den Wyngaert, Neriman Yilmaz, Andrey Yurkov, Urmas Kõljalg, Kessy Abarenkov

Supplementary material 3 The metadata annotations for the sequences that were found in the same SHs as sequences from the built environment Data type: molecular data This is an open access article distributed under the terms of the Creative Commons Attribution License (CC BY 4.0), which permits unrestricted use, distribution, and reproduction in any medium, provided the original author and source are credited.R. Henrik Nilsson, Andy F. S. Taylor, Rachel I. Adams, Christiane Baschien, Johan Bengtsson-Palme, Patrik Cangren, Claudia Coleine, Heide-Marie Daniel, Sydney I. Glassman, Yuuri Hirooka, Laszlo Irinyi, Reda Iršėnaitė, Pedro M. Martin-Sanchez, Wieland Meyer, Seung-Yoon Oh, Jose Paulo Sampaio, Keith A. Seifert, Frantisek Sklenář, Dirk Stubbe, Sung-Oui Suh, Richard Summerbell, Sten Svantesson, Martin Unterseher, Cobus M. Visagie, Michael Weiss, Joyce Woudenberg, Christian Wurzbacher, Silke Van den Wyngaert, Neriman Yilmaz, Andrey Yurkov, Urmas Kõljalg, Kessy Abarenkov

Supplementary material 4 The interactive Krona chart associated with Figure 2 Data type: molecular data This is an open access article distributed under the terms of the Creative Commons Attribution License (CC BY 4.0), which permits unrestricted use, distribution, and reproduction in any medium, provided the original author and source are credited.R. Henrik Nilsson, Andy F. S. Taylor, Rachel I. Adams, Christiane Baschien, Johan Bengtsson-Palme, Patrik Cangren, Claudia Coleine, Heide-Marie Daniel, Sydney I. Glassman, Yuuri Hirooka, Laszlo Irinyi, Reda Iršėnaitė, Pedro M. Martin-Sanchez, Wieland Meyer, Seung-Yoon Oh, Jose Paulo Sampaio, Keith A. Seifert, Frantisek Sklenář, Dirk Stubbe, Sung-Oui Suh, Richard Summerbell, Sten Svantesson, Martin Unterseher, Cobus M. Visagie, Michael Weiss, Joyce Woudenberg, Christian Wurzbacher, Silke Van den Wyngaert, Neriman Yilmaz, Andrey Yurkov, Urmas Kõljalg, Kessy Abarenkov
