# Supplementary material for: Taxonomic annotation of public fungal ITS sequences from the built environment – a report from an April 10–11, 2017 workshop (Aberdeen, UK)
Source: MycoKeys. 2018 Jan 8;(28):65–82. doi: 10.3897/mycokeys.28.20887 (PMC5804120; doi:10.3897/mycokeys.28.20887)
Supplement: Supplementary material 1 — The sequences renamed during the workshop. The INSDC accession number, the original INSDC name, and the new UNITE name are shown [file mycokeys-28-065-s004.html]

Javascript must be enabled to view this page.

magnitude
 7474
 7416
 3964
 75
 3
 1
 1
 15
 1
 54
 23
 23
 4
 4
 1261
 2
 2
 62
 10
 8
 528
 36
 609
 2
 2
 1384
 1246
 1
 1
 1
 89
 42
 3
 1
 889
 12
 21
 50
 1
 51
 7
 1
 11
 33
 2
 696
 4
 139
 137
 2
 20
 1
 1
 1
 7
 5
 1
 4
 1
 1
 8
 8
 3
 1
 2
 157
 157
 996
 1
 1
 11
 1
 4
 1
 2
 1
 2
 446
 32
 1
 30
 19
 112
 12
 4
 5
 18
 1
 4
 1
 8
 133
 5
 61
 7
 6
 1
 60
 1
 2
 57
 16
 16
 262
 21
 4
 60
 142
 7
 28
 82
 82
 1
 1
 36
 36
 12
 10
 1
 1
 52
 52
 10
 10
 6
 6
 6
 46
 3
 3
 43
 43
 2404
 2404
 2404
 1
 1
 1
 1
 55
 3
 3
 3
 51
 51
 51
 1
 1
 1
 1
 1
 1
 1
 1
 1
 1
 1
